# Mitral regurgitation increases systolic strains in remote zone and worsens left ventricular dyssynchrony in a swine model of ischemic cardiomyopathy

**DOI:** 10.3389/fcvm.2024.1397079

**Published:** 2024-05-28

**Authors:** Yuta Kikuchi, Daisuke Onohara, Michael Silverman, Chase L. King, Stephanie K. Tom, Riya Govin, Robert A. Guyton, Muralidhar Padala

**Affiliations:** ^1^ Cardiothoracic Research Laboratories, Carlyle Fraser Heart Center at Emory University Hospital Midtown, Atlanta, GA, United States; ^2^ Department of Surgery, Division of Cardiothoracic Surgery, Emory University School of Medicine, Atlanta GA, United States; ^3^ Department of Biomedical Engineering, Georgia Institute of Technology, Atlanta, GA, United States

**Keywords:** ischemic mitral regurgitation, volume overload, longitudinal strain, circumferential strain, dyssynchrony

## Abstract

**Background:**

Ischemic mitral regurgitation (IMR) imposes volume overload on the left ventricle (LV), accelerating adverse LV remodeling. In this study, we sought to investigate the impact of volume overload due to IMR on regional myocardial contractile mechanics.

**Methods:**

Ten Yorkshire swine were induced with myocardial infarction (MI) by occluding the left circumflex coronary artery (LCx). Cardiac MRI was performed at baseline (BL) and 2.5 months (2.5M) post-MI. IMR was quantified with epicardial echocardiography 3 months post-MI. The animals were then assigned to 2 groups: no/mild MR (nmMR, *n* = 4) and moderate/severe MR (msMR, *n* = 6). MRI images were analyzed to assess infarction size, end-diastolic and end-systolic volume (EDV and ESV, respectively), ejection fraction (EF), longitudinal strain (LS), circumferential strain (CS), and systolic dyssynchrony index (SDI). The myocardial region was divided into infarction, border, and remote zones based on the LCx-supplied region.

**Results:**

There was no difference in the infarction size. Group-wise comparison of LS and CS between BL and 2.5M demonstrated that LS and CS in the infarction zone and the border zone decreased at 2.5M in both groups. However, LS and CS in the remote zone were elevated only in the msMR group (LS: −9.81 ± 3.96 vs. −12.58 ± 5.07, *p* < 0.01; CS; −12.78 ± 3.81 vs. −16.09 ± 3.33, *p* < 0.01) at 2.5M compared to BL. The SDI of CS was significantly elevated in the msMR group (0.1255 vs. 0.0974, *p* = 0.015) at 2.5M compared to BL.

**Conclusions:**

Elevated LS and CS in the remote zone were observed in moderate/severe MR and ventricular dyssynchrony. These elevated cardiac strains, coupled with ventricular dyssynchrony, may contribute to the progression of MR, thereby accelerating heart failure.

## Introduction

Mitral regurgitation (MR) imposes volume overload and adverse remodeling on the ventricular myocardium, leading to left ventricular (LV) dilatation and a spherical heart shape, which decreases pumping efficiency, in the long term (1–3). Functional MR, a subtype of MR, often occurs in conjunction with ischemia, known as ischemic mitral regurgitation (IMR). IMR complicates up to 50% of patients with post-myocardial infarction (MI) and contributes to increased hospitalization and mortality (4, 5). In considering the pathophysiological aspects of MR, the volume burden during the diastolic phase has been thoroughly investigated. However, limited research has addressed the impact of volume overload on the biomechanics of LV contractility in IMR.

Cardiac Strains have been used to elucidate the LV contractile mechanics, given that LV contraction involves three vectors based on the myocardial orientation: longitudinal, circumferential, and radial strains (6). Global longitudinal strain (GLS) is acknowledged as a better predictor of LV function than ejection fraction (EF) due to its less volume load dependence (7). However, in IMR, a regional evaluation, not just the GLS, is required for accurate characterization of the myocardium. This is attributed to the irregular pattern of the ischemic area, necessitating a comprehensive assessment for a thorough understanding of myocardial dynamics. Therefore, it is beneficial to observe the individual myocardial responses to volume overload within the infarction, border, and remote zones.

In addition, the ventricular dyssynchrony observed in patients with IMR is a critical factor in the progression of heart failure due to loss of pumping efficiency. This dyssynchrony can also worsen MR by causing a discoordination of the papillary muscles and reducing the closing force of the LV (8). Several studies demonstrated that resynchronization therapy effectively reduced MR, especially if the patients have left bundle branch block (9–11). These findings further support the association between MR deterioration and dyssynchrony. However, there is no report regarding dyssynchrony using cardiac strains in the setting of IMR as well as regional strain evaluation. Assessing regional variations in cardiac strains could facilitate the early detection of dyssynchrony.

In this study, we hypothesized that regional longitudinal and circumferential strains, as well as their dyssynchrony, would vary corresponding to the severity of MR based upon the extent of volume load. To investigate this hypothesis, we utilized Yorkshire swine with IMR after similar-sized MI and analyzed the cardiac strains using cardiac MRI.

## Materials and methods

### Experimental design

**Figure 1:**
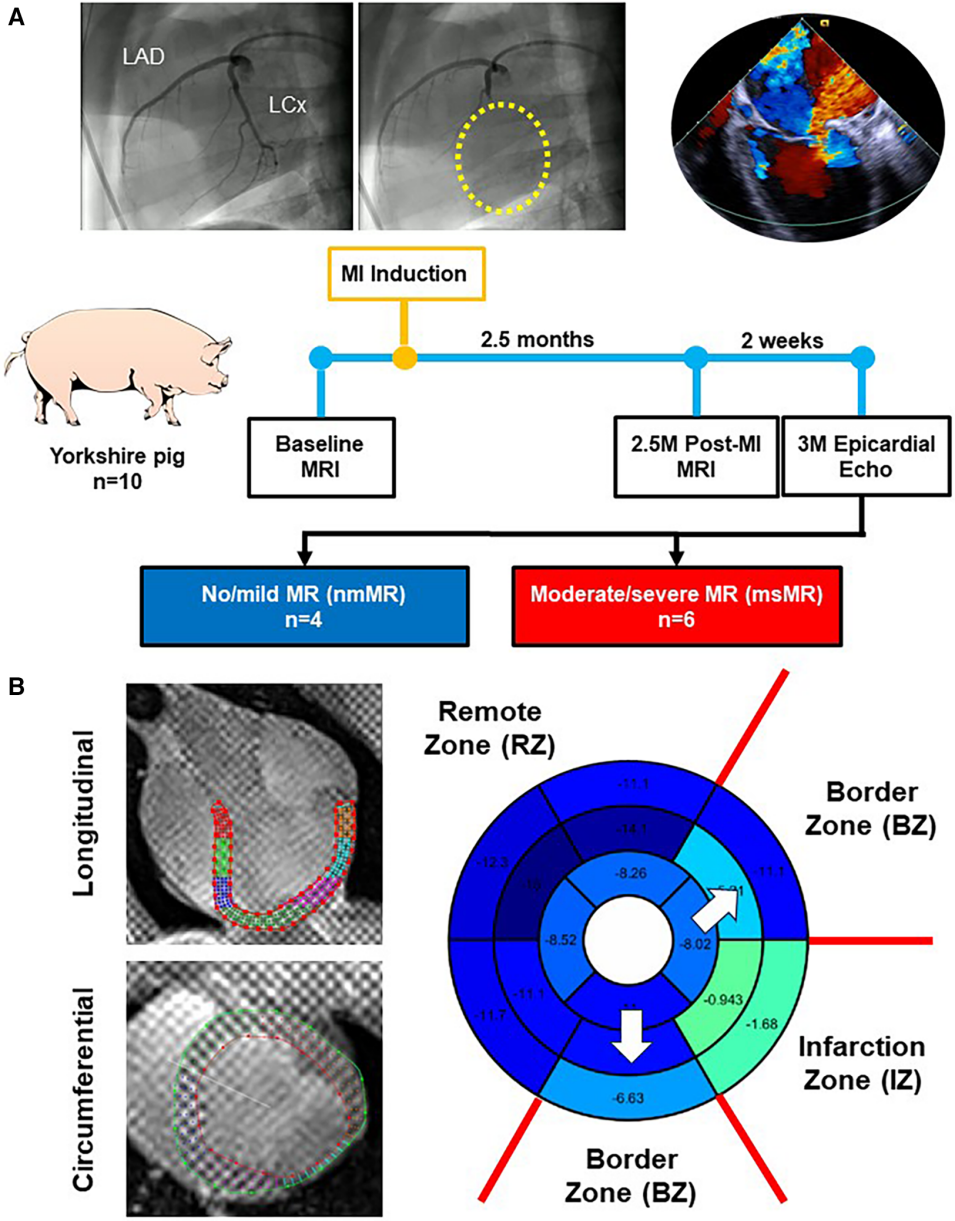
(**A**) Experimental design. (**B**) Representative images of the long-axis and short-axis view with contour using Segment software. The bull's eye is an example of peak strain distribution after analyzing and zone classification of myocardial regions in this study. MRI, magnetic resonance image; MI, myocardial infarction; MR, mitral regurgitation.

Figure 1A depicts the experimental design in this study. Ten Yorkshire swine underwent baseline (BL) cardiac magnetic resonance imaging (MRI). Subsequently, myocardial infarction (MI) was induced by occluding left circumflex coronary artery branches (LCx). Cardiac MRI was repeated at 2.5 months (2.5M) post-MI, and epicardial echocardiography was performed through a left mini thoracotomy to evaluate MR at 3 months post-MI.

### Animal acquisition and housing

All procedures were authorized by the Institutional Animal Care and Use Committee, adhering to the guidelines outlined in the PHS policy on humane care and use of laboratory animals issued by the National Institutes of Health in Bethesda, MD. Procedures were performed following the guidelines of the National Institutes of Health for the use of animals and pain control. A total of 10 Yorkshire swine, weighing 35–50 kg each, were obtained from a USDA-approved farm (Valley Brook Farm, GA or Palmetto Farms, SC).

### Animal preparation for procedures

The swine were subjected to overnight fasting in preparation for the experimental procedures. On the day of the procedure, the animals were sedated with an appropriate dose of Telazol (2–8.8 mg/kg, IM), followed by weighing. After the initiation of mechanical ventilation, general anesthesia was induced with approximately 2% isoflurane in 100% oxygen and was maintained throughout the procedure. In addition, Cefazolin (22–25 mg/kg, IV), Rimadyl (2 mg/kg, IM), and Buprenex (0.0075–0.01 mg/kg, IM) were administered preoperatively. Propofol (1–3 mg/kg, IV) was used as supplementary anesthetic support as needed. Amiodarone (0.5 mg/kg, slow DIV) was continuously administered to prevent ventricular arrhythmias during the procedures except for MRI. Phenylephrine and Dobutamine were given as required to maintain a mean arterial pressure within the range of 60–70 mmHg.

### Cardiac MRI and analysis

The swine were sedated by the method outlined above and placed in a supine position on the bed of a Prisma Fit MRI (Siemens Healthineers, Munich, Germany), which has a three-tesla magnetic field strength. All imaging was conducted using an ultra-flex large coil with 18 channels. First, long-axis cine images, including 2 Chamber (CH), 3 Chamber (CH), and 4 Chamber (CH) views, were acquired. Subsequently, short-axis cine images were obtained, spanning from the apex to the base of the LV. Tagging images were then acquired in both long and short-axis views. Lastly, late gadolinium enhancement (LGE) imaging was performed.

For LV volume function scans, gradient echo cine images were conducted with an in-plane resolution of 1.6 mm and a slice thickness of 6 mm. Retrospective ECG gating was employed with 24 frames reconstructed over the cardiac cycle. Images were acquired during suspended breathing. The tagging images for strain analysis were the same as above but grid tags were employed with 5 mm tag spacing. For the LGE to clarify the infarction area, Phase-sensitive inversion recovery (PSIR) with a gradient echo with an in-plane resolution of 1.6 mm and a slice thickness of 6 mm. The delay time and inversion time were based on the review of cine images and TI scout images, respectively. Imaging was acquired with an in-plane resolution of 1.6 mm and a slice thickness of 6 mm.

The infarct area was determined using LGE images of the LV short-axis view, calculated as the ratio of the infarct region's circumference to that of the entire LV. Image J software was utilized for this measurement in the Base, Mid, and Apical views (12). For analysis of longitudinal strain (LS) and circumferential strain (CS), Segment software (Medviso, Lund, Sweden) was used (13–15). The long-axis views, including 2 CH, 3 CH, and 4 CH views, were used for LS, while the short-axis views, including Base, Mid, and Apical views, were used for CS. Due to limitations in image resolution in both strains during the diastolic phase, our analysis focused solely on the systolic phase, and the radial strain was eliminated from this study. Dyssynchrony was assessed by calculating the standard deviation of ‘Time to Peak’ in both LS and CS, which is called the systolic dyssynchrony index (SDI) (16, 17).

The myocardial region was divided into three zones based on the LCx-supplied region to investigate the differences in strains and dyssynchrony. The infarction zone (IZ) includes basal inferolateral, and mid inferolateral regions. The border zone (BZ) includes basal anterolateral, basal inferior, mid anterolateral, mid inferior, apical lateral, and apical inferior regions. The remote zone (RZ) includes basal anterior, basal anteroseptal, basal inferoseptal, mid anterior, mid anteroseptal, mid inferoseptal, apical anterior, and apical septal regions (Figure 1B).

### Myocardial infarction procedure

Both the groins and the left chest surgical areas were shaved and then scrubbed with alcohol and Betadine. First, the right femoral artery was isolated with a cut-down, and an 8Fr arterial sheath (SuperSheath®, Boston Scientific, Natick, MA) was placed with the Seldinger technique for blood pressure monitoring and catheterization. Subsequently, a 6Fr pig-tail catheter was inserted via the arterial sheath to enter the left ventricle (LV) for ventriculography (AP angle and 60° LAO + 15° CAU) to locate the posteromedial papillary muscle (PMPM). A 6Fr hockey stick catheter (Vista Britetip®, Cordis, Miami Lakes, FL) was inserted via the arterial sheath and engaged at the ostium of the right coronary artery, then engaged at the ostium of the left coronary artery for angiography (AP angle and 60° LAO + 15° CAU) to identify the distribution of coronary arteries. A coronary artery occlusion procedure was undertaken using a suitable-sized coronary angioplasty balloon (2–3 mm*8 mm, Trek over the wire, Abbott Vascular®), which was carefully advanced over a guidewire into the LCx branches, the primary blood supplier to the PMPM. Following balloon inflation at the target site, typically just after the obtuse marginal branch, the guide wire was retracted. 100% ethanol (1.5 ml in each vessel, E7023, Sigma-Aldrich, USA) was gradually injected into the artery through the central lumen of the balloon for a duration of 10 min. The balloon remained inflated for an additional 15 min to induce thrombus formation while preventing the backflow of ethanol into the other left coronary branches. This step was repeated for each target vessel as necessary. A final angiogram and echocardiogram were then performed to confirm permanent occlusion of the arteries, a reduction in ejection fraction to less than 40%, and the presence of mitral regurgitation.

### Epicardial echocardiography

A transesophageal ultrasound probe (Z6Ms, 3–6.3 MHz, Siemens SC2000 PRIME, Seattle, WA) was placed on the left atrial roof to obtain 3CH view, which includes the left atrium (LA), left ventricle (LV) and left ventricular outflow tract (LVOT) through the left mini-thoracotomy. Velocity vector imaging (VVI) software was utilized to calculate endo-diastolic volume (EDV), endo-systolic volume (ESV), stroke volume (SV), and ejection fraction (EF). Mitral regurgitation (MR) was evaluated with color and continuous wave Doppler spectrum. Effective regurgitant orifice area (EROA) was calculated with the proximal isovelocity surface area (PISA) method using software mounted on the echo. Regurgitant volume (Rvol) and regurgitant fraction (RF) were then calculated using the following formula. MR severity was quantified based on the 2020 ACC/AHA guidelines (18).

Rvol=EROA∗Velocitytimeintegral(VTI)ofMR

RF=Rvol/LVstrokevolume

### Statistical analysis

GraphPad Prism 10 (GraphPad Prism Software Inc., San Diego, CA) was used for all statistical analyses and graphs in this study. Continuous variables were tested with the Shapiro-Wilk normality test to determine the distribution. Normal distribution variables were presented as mean ± SD and compared using the paired or unpaired *t*-tests. For non-normal distribution variables, median and interquartile range (IQR) were presented, and comparisons were performed using the Mann-Whitney or Wilcoxon test. The SDI was compared with the F test. Statistical significance was achieved if the *p*-value was less than 0.05.

It is important to note that our study did not incorporate an analysis of inter- and intra-observer variability. MR quantification was conducted by three experienced individuals with a comprehensive understanding of echocardiographic findings concurrently utilizing the PISA method calculation on the Siemens echo system. Additionally, strain analysis was conducted by a singular investigator (Yuta Kikuchi) employing an automated strain analysis application. Hence, we believe that the influence of intra- and inter-observer variability on our study outcomes is deemed minimal.

## Results

### Infarction area, LV function/geometry change, and MR quantification

**Figure 2:**
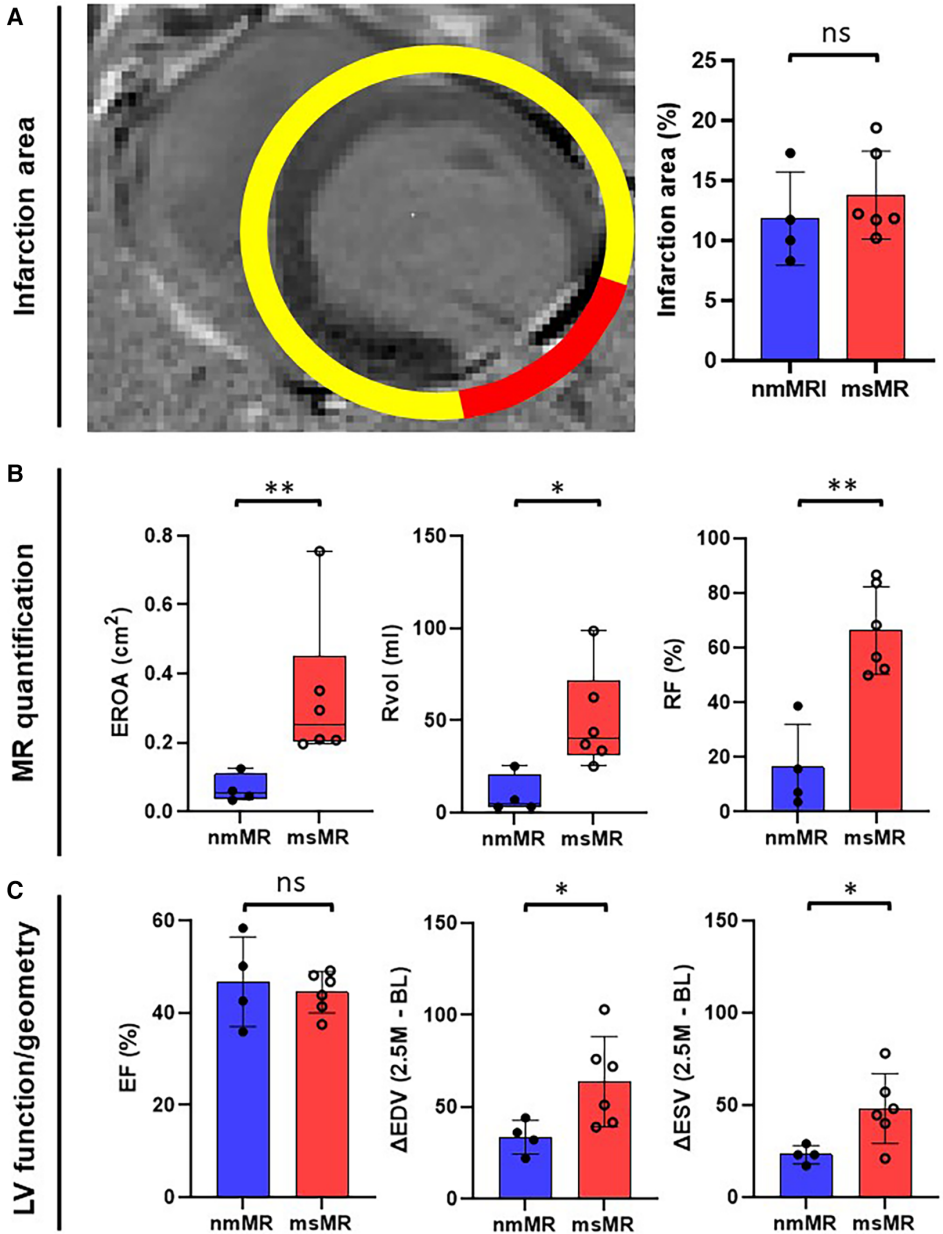
(**A**) The representative MRI image of gadolinium enhancement with clear lateral infarction. There is no difference in infarction size at 2.5M. (**B**) MR quantification with EROA, Rvol, and RF. They are greater in msMR at 2.5M, meaning that the classification is effective. (**C**) There is no difference in EF. However, the volume change (ΔEDV and ΔESV) from BL to 2.5M is greater in msMR. *p < 0.05, **p < 0.01. MRI, magnetic resonance image; M, month; MR, mitral regurgitation; EROA, effective regurgitation orifice area; Rvol, regurgitant volume; RF, regurgitant fraction; EF, ejection fraction; EDV, endo diastolic volume; ESV, endo systolic volume.

Figure 2 depicts a representative image of LGE with clear inferolateral infarction and the differences in infarction area (Figure 2A), MR quantification (Figure 2B), and LV function/geometry change (Figure 2C). There is no significant difference in the infarction area between nmMR (11.85 ± 3.89%) and msMR (13.79 ± 3.65%). EROA {0.2520 cm^2^ (0.2047, 0.4524) vs. 0.0524 cm^2^ (0.0358, 0.1082), *p* < 0.01}, Rvol {40.36 ml (31.54, 71.56) vs. 5.05 ml (3.08, 20.66), *p* = 0.02} and RF (66.26 ± 16.01% vs. 16.31 ± 15.78%, *p* < 0.01) were significantly greater in msMR at 3M, which indicates that MR was accurately classified. There are no significant differences in ejection fraction (EF), endo-diastolic volume (EDV), and endo-systolic volume (ESV) at BL and 2.5M, but ΔEDV (63.75 ± 24.61 ml vs. 33.50 ± 9.15 ml, *p* = 0.049) and ΔESV (48.08 ± 18.90 ml vs. 23.00 ± 4.90 ml, *p* = 0.034), the volume changes from BL to 2.5M, were significantly higher in msMR (Table 1).

**Table 1:** Characteristics at 2.5M with MRI and 3M with echo (MR quantification).

	No/mild MR (*n* = 4)	Moderate/ severe MR (*n* = 6)	*p* Value
Infarction ratio, %	11.85 ± 3.89	13.79 ± 3.65	0.45
End-diastolic volume, mL	127 ± 28.6	149 ± 27.8	0.26
ΔEDV (EDV_2.5M_ - EDV_Baseline_)	33.5 ± 9.2	63.8 ± 24.6	**0** **.** **049**
End-systolic volume, mL	67.7 ± 19.6	83.5 ± 20.7	0.26
ΔESV (ESV_2.5M_ - ESV_Baseline_)	23 ± 4.9	48.1 ± 18.9	**0**.**03**
Ejection fraction, %	46.73 ± 9.70	44.44 ± 4.46	0.62
MR quantification with Echography			
Effective regurgitant orifice area, cm^2^	0.052 (0.036–0.108)	0.252 (0.205–0.452)	**<0.01**
Regurgitant volume, mL	5.05 (3.08–20.66)	40.36 (31.54–71.56)	**0**.**02**
Regurgitant fraction, %	16.31 ± 15.78	66.26 ± 16.01	**<0.01**

Bold values indicate statistical significance, *p* < 0.05.

### Longitudinal strain

**Figure 3:**
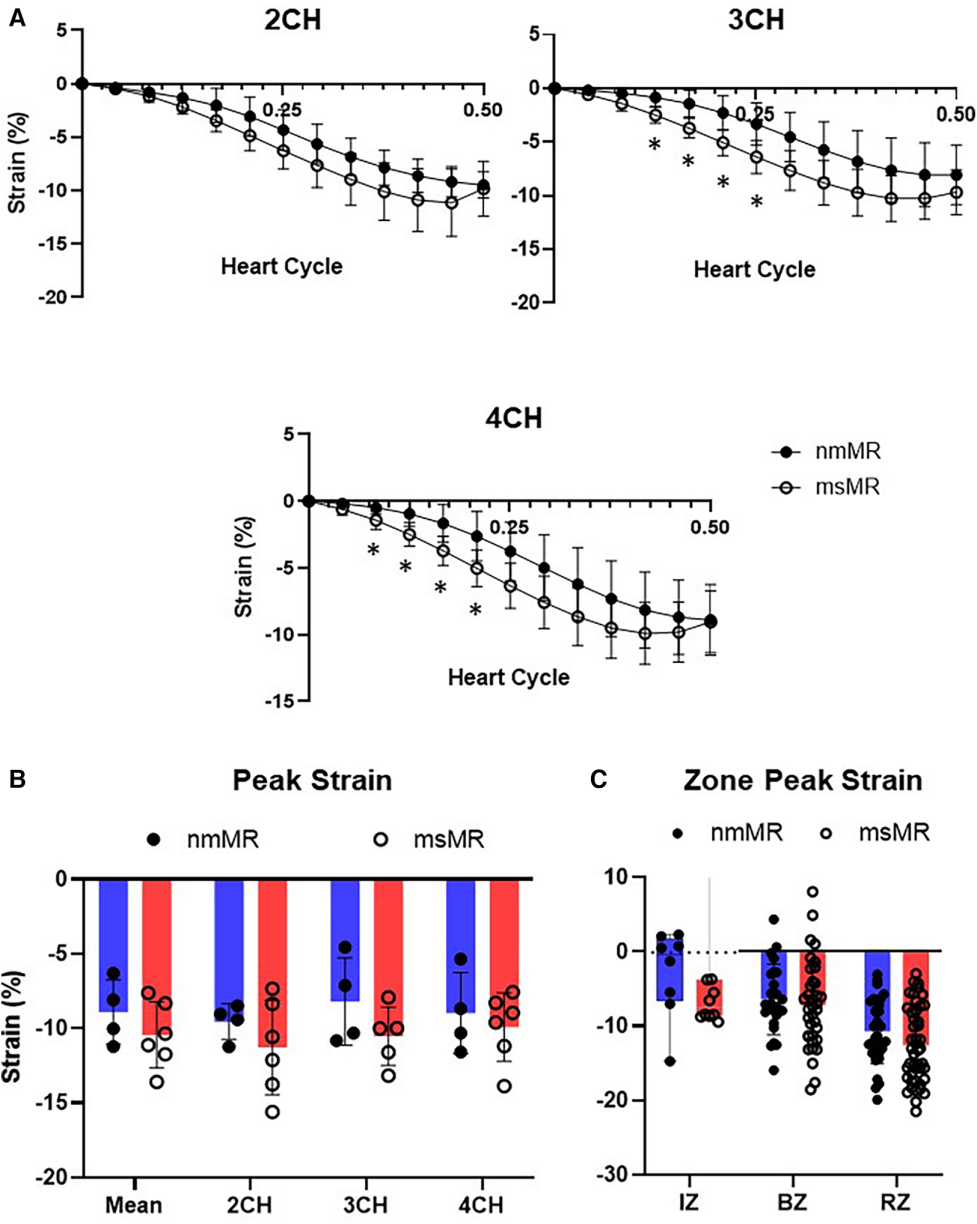
Comparison of LS at 2.5M between nmMR and msMR. (**A**) Time course of LS during end-diastole to end-systole in each chamber view. Some early higher LS is observed in 3CH and 4CH. (**B**) Peak strain in each chamber and mean of them with no significant difference. (**C**) Peak strain in each zone. Significant higher LS is observed in RZ in msMR. *p < 0.05. LS, longitudinal strain; CH, chamber; IZ, infarction zone; BZ, border zone; RZ, remote zone.

At BL, the time course of strain in 2 CH, 3 CH, and 4 CH views during the systolic heart cycle was comparable. Also, there were no differences in the peak strain in each chamber view, their mean values, and the zone strain (Supplementary Figure 1). At 2.5M, there was also no difference in the time course of strain in 2 CH views. However, some early elevated LS was observed in 3 CH and 4 CH views in msMR compared to nmMR (Figure 3A). The differences in the peak strain across each chamber view, their mean value, and the zone strain were not statistically significant, as illustrated in Figures 3B,C.

**Figure 4:**
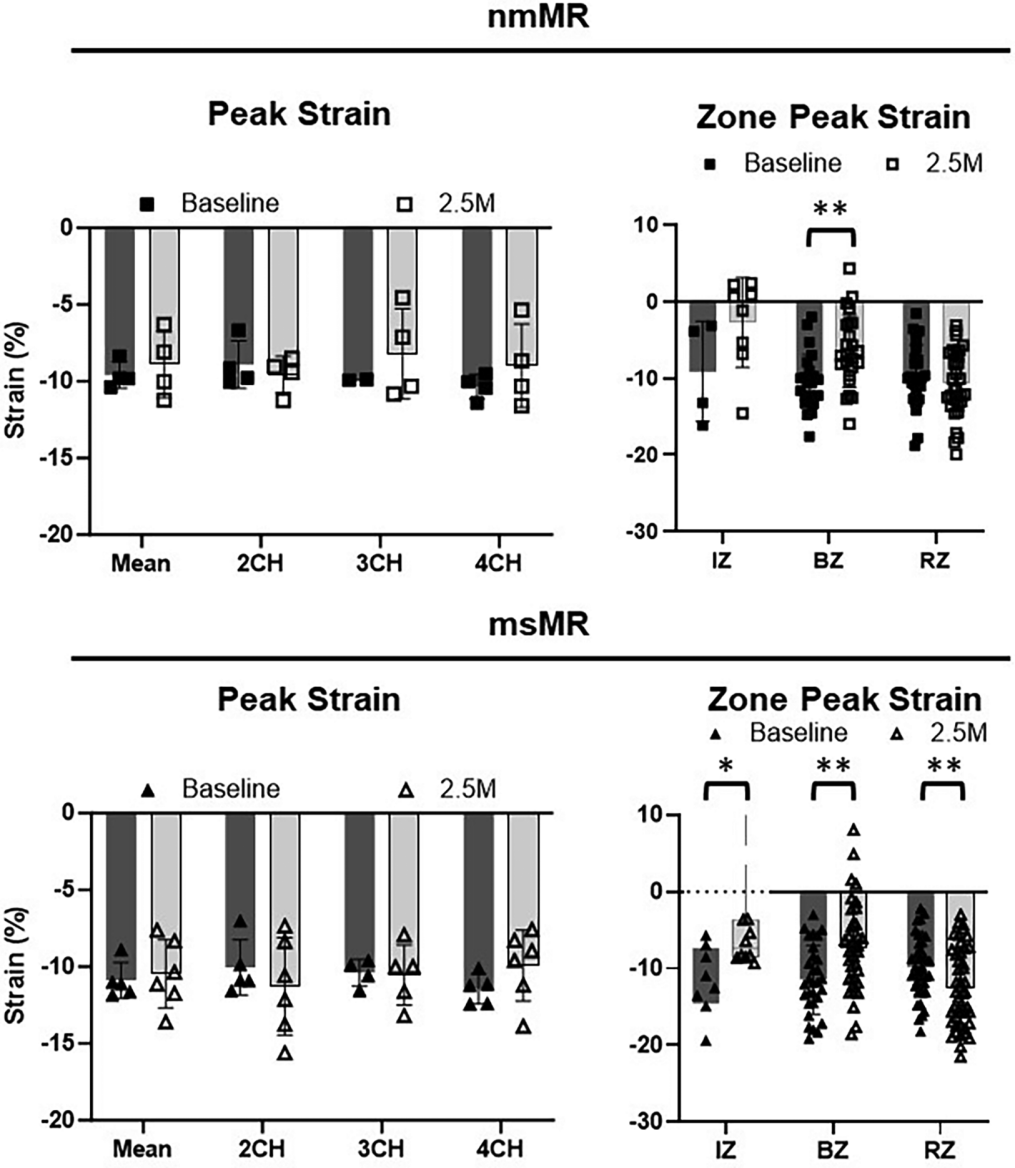
Ls differences between BL and 2.5M in each group. There are no differences in peak strain in each chamber and mean of them. Lower LS in IZ is observed in both groups and there is significantly higher LS in RZ only in msMR at 2.5M compared to BL. *p < 0.05, **p < 0.01. LS, longitudinal strain; M, month; IZ, infarction zone; BZ, border zone; RZ, remote zone.

Comparing LS between BL and 2.5M in each group (Figure 4, Table 2), there were no significant differences in the peak strain in each chamber or their mean. However, LS in IZ tended to decrease in nmMR and significantly decreased in msMR at 2.5M {nmMR; −9.10 ± 6.56 vs. −2.73 ± 5.87, *p* = 0.16, msMR; −11.73 (−14.52, −7.31) vs. −7.33 (−8.51, −3.60), *p* < 0.05} due to MI. Also, LS in BZ significantly decreased in both groups {nmMR; −10.52 ± 3.62 vs. −6.43, *p* < 0.01, msMR; −11.46 ± 4.47 vs. −7.22 ± 5.87, *p* < 0.01}. Interestingly, LS in RZ was significantly elevated *only* in msMR (−9.81 ± 3.96 vs. −12.58 ± 5.07, *p* < 0.01).

**Table 2:** Longitudinal and circumferential strain at baseline and 2.5 months.

Longitudinal strain	Infarction zone			Border zone			Remote zone		
	Baseline	2.5M	*p* value	Baseline	2.5M	*p* value	Baseline	2.5M	*p* value
nmMR	−9.10 ± 6.56	−2.73 ± 5.87	0.12	−10.52 ± 3.62	−6.43 ± 4.75	**<0.01**	−9.61 ± 3.97	−10.72 ± 4.34	0.21
msMR	−11.53 ± 4.51	−7.33 (−8.51, −3.60)	**0.02**	−11.46 ± 4.47	−7.22 ± 5.87	**<0.01**	−9.90 ± 4.06	−12.58 ± 5.07	**<0.01**
*p* value	0.72	0.15		0.79	0.57		0.99	0.13	
Circumferential strain	Infarction zone			Border zone			Remote zone		
	Baseline	2.5M	*p* value	Baseline	2.5M	*p* value	Baseline	2.5M	*p* value
nmMR	−12.98 ± 1.81	−4.37 ± 6.62	**<0.01**	−12.01 (−15.50, −9.69)	−9.05 ± 3.85	**<0.01**	−13.37 ± 4.15	−13.13 ± 3.40	0.26
msMR	−14.66 (−16.20, −13.44)	−6.74 ± 5.62	**<0.01**	−13.62 (−14.96, −11.67)	−10.13 ± 7.01	0.05	−12.78 ± 3.81	−16.09 ± 3.33	**<0.01**
*p* value	0.41	0.34		0.62	0.34		0.76	**<0.01**	

Bold values indicate statistical significance, *p* < 0.05.

### Circumferential strain

**Figure 5:**
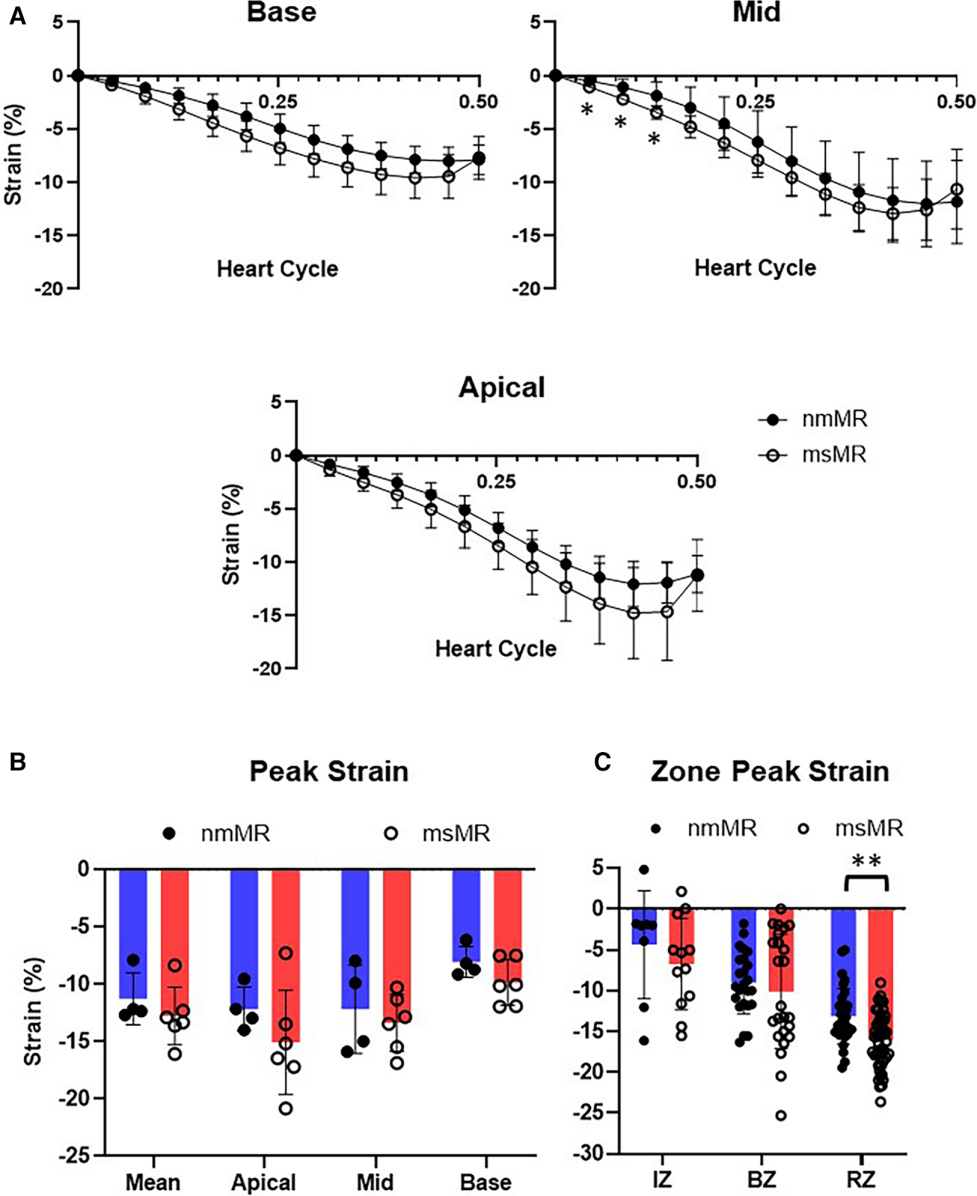
Comparison of CS at 2.5M between nmMR and msMR. (**A**) Time course of CS during one heart cycle in each chamber view. Some early higher CS is observed in 3CH. (**B**) Peak strain in each chamber and mean of them with no significant difference. (**C**) Peak strain in each zone. Significant higher CS is observed in RZ in msMR. *p < 0.05, **p < 0.01. CS, circumferential strain; IZ, infarction zone; BZ, border zone; RZ, remote zone.

Each section (Base, Mid, and Apical) showed no differences in the CS time course at BL. Similarly, there were no differences in peak strain in each section, their mean values, and the zone strain (Supplementary Figure 2). At 2.5M, some early higher CS were noted in Mid in msMR compared to nmMR, but no other differences in the CS time course were observed between both groups (Figure 5A). Additionally, there was also no significant difference in peak strain in each section and the mean of them (Figure 5B). However, higher CS was observed in RZ in msMR (−13.13 ± 3.40 vs. −16.09 ± 3.33, *p* < 0.01) (Figure 5C).

**Figure 6:**
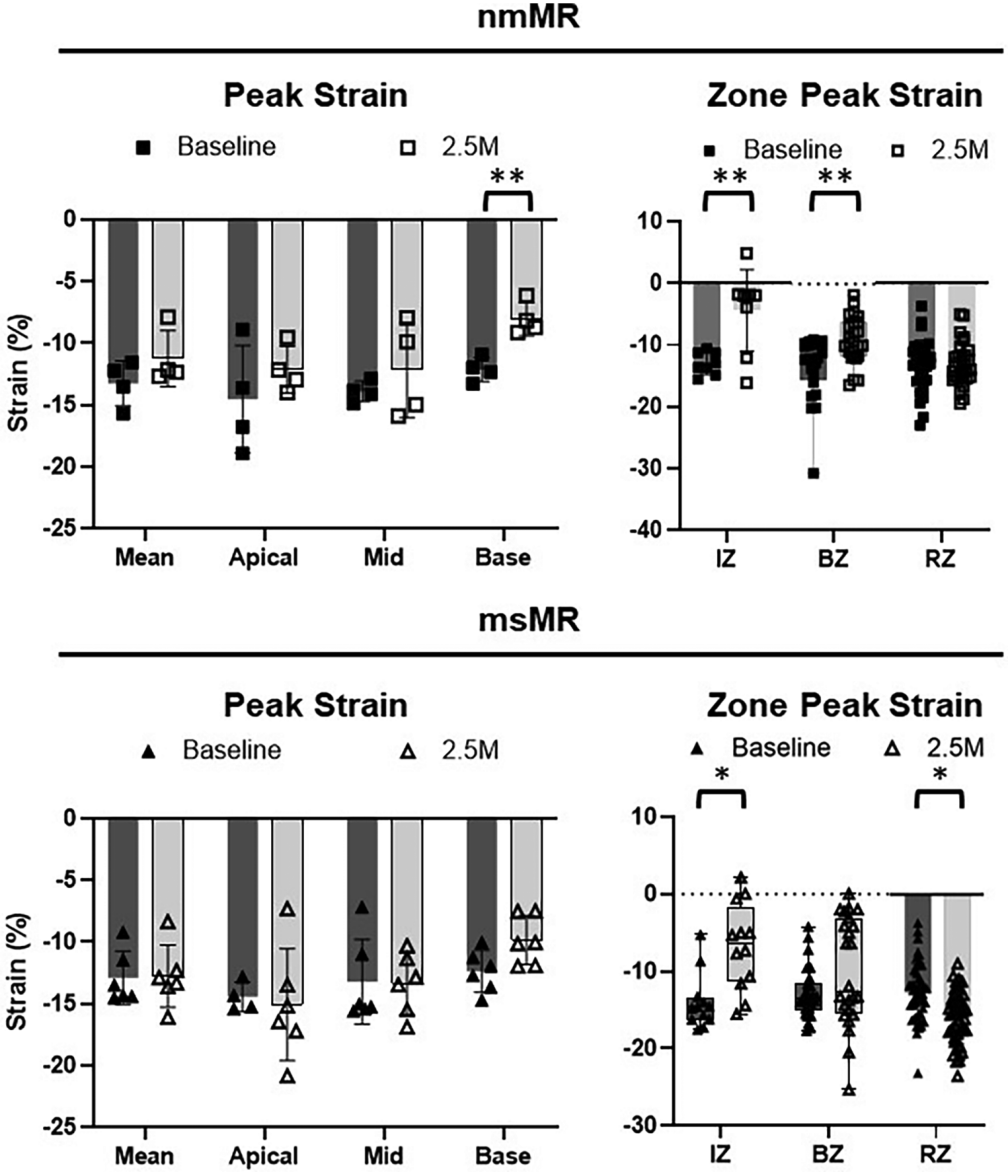
Cs differences between BL and 2.5M in each group. There are significantly lower CS in Base at 2.5M in nmMR. Lower CS in IZ is observed in both groups and there is significantly higher LS in RZ only in msMR at 2.5M compared to BL. *p < 0.05, **P < 0.01. CS, circumferential strain; BL, baseline; M, month; IZ, infarction zone; BZ, border zone; RZ, remote zone.

Comparing CS between BL and 2.5M in each group (Figure 6, Table 2), a lower CS in Base was observed in nmMR at 2.5M (nmMR; −8.05 ± 1.33 vs. −12.13 ± 0.97, *p* < 0.01). In the comparison of CS in each zone, similar to LS, both groups showed a decrease in IZ at 2.5M {nmMR; −12.98 ± 1.81 vs. −4.37 ± 6.62, *p* < 0.01, msMR; −14.66 (−16.20, −13.44) vs. −6.33 (−11.35, 1.69), *p* < 0.01}. Also, CS in BZ significantly decreased in nmMR at 2.5M {−12.01 (−15.50, −9.69) vs. −9.38 (−11.67, −6.18), *p* < 0.01}. CS in RZ was increased *only* in msMR at 2.5M (−12.78 ± 3.81 vs. −16.09 ± 3.33, *p* < 0.01) as the same with LS.

### Correlation between strains and EDV

**Figure 7:**
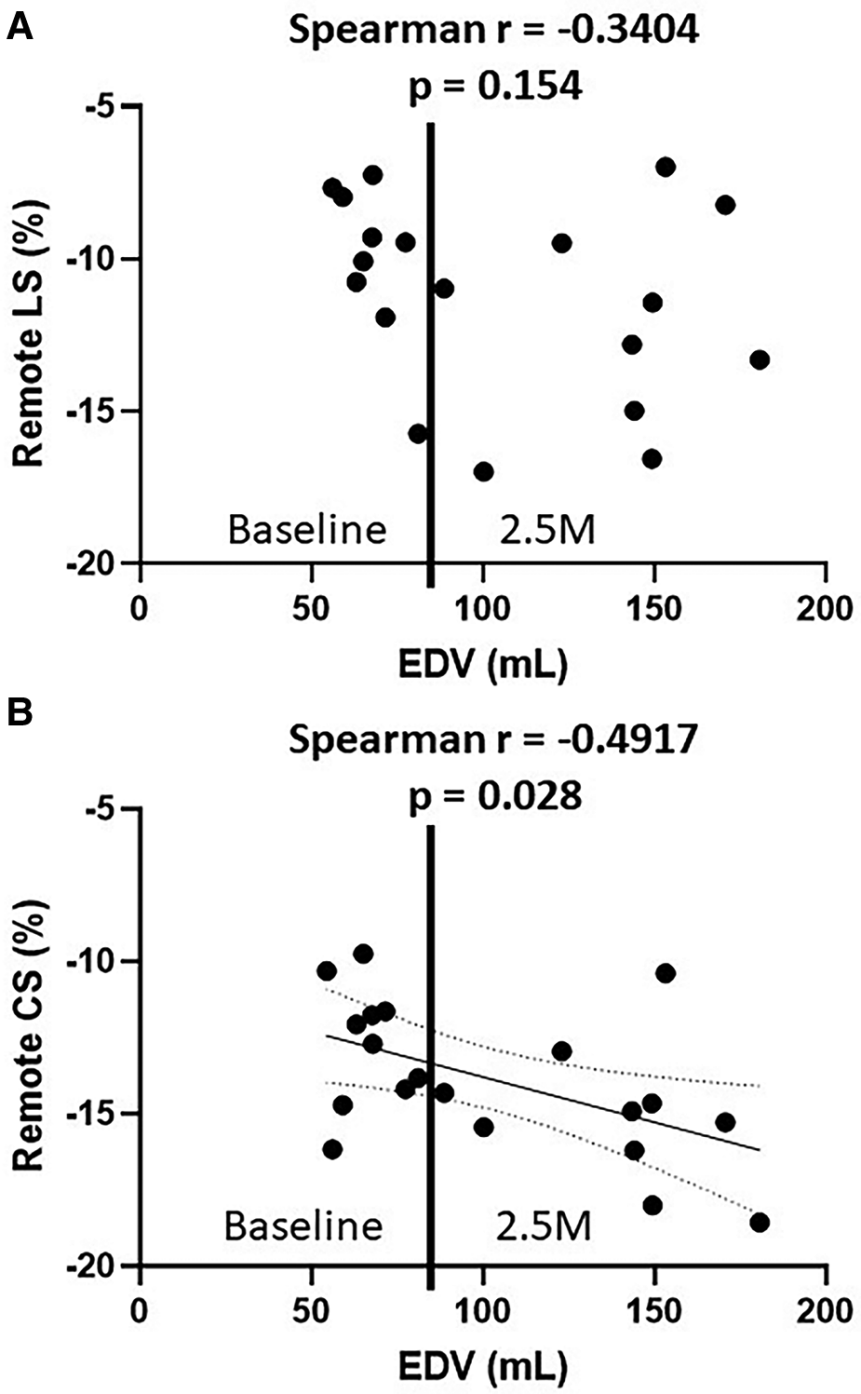
The relationship between EDV and remote LS (**A**), EDV and remote CS (**B**) EDV, end-diastolic volume; LS, longitudinal strain; CS, circumferential strain.

The correlation between EDV and cardiac strains in the remote zone was assessed using all baseline and 2.5 months data (*n* = 20). As shown in Figure 7, CS in the remote zone was weakly correlated with EDV (Spearman r = −4.917, *p* = 0.03), indicating that volume overload elevated the remote zone CS following Frank-Starling law, although LS showed no correlation with EDV (Spearman r = −0.3404, *p* = 0.15).

### Dyssynchrony

The SDI was compared between BL and 2.5M in each group. No significant difference was observed in nmMR. However, LS-SDI tended to be higher (0.148 vs. 0.122, *p* = 0.075) and CS-SDI was significantly elevated at 2.5M (0.126 vs. 0.097, *p* = 0.017) in msMR, indicating that there was greater dyssynchrony in msMR compared to nmMR (Table 3).

**Table 3:** Dyssynchrony Index.

	no/mild MR *n* = 4			Moderate/severe MR *n* = 6		
	Baseline	2.5M	*p* Value	Baseline	2.5M	*p* Value
LS-SDI	0.1303	0.1293	0.94	0.122	0.1478	0.075
CS-SDI	0.08575	0.09387	0.48	0.09741	0.1255	**0**.**017**

Bold values indicate statistical significance, *p* < 0.05.

## Discussion

Ischemic mitral regurgitation (IMR) is a pathophysiological condition resulting from ischemic injury to the myocardium, specifically affecting the left ventricular inferolateral wall. This injury often stems from coronary artery disease, leading to MI. The ischemic damage can cause alterations in the geometry and function of the LV, known as ventricular remodeling. This remodeling involves dilation, transformation into a more spherical shape, and altered contractility of the LV, which causes mitral regurgitation due to the tethering of the mitral valve. Understanding the complex interplay between ventricular remodeling, dysfunction of the mitral valve apparatus, and resultant hemodynamic compromise is critical for the effective management of IMR.

Ejection fraction (EF) is a widely recognized parameter for assessing cardiac function and remodeling. However, its limitations have been highlighted in the context of modern cardiology, where the demands for detecting subclinical left ventricular dysfunction and recognizing changes in left ventricular function are more stringent (19–22). Therefore, myocardial strain has been increasingly recognized as a more sensitive parameter for evaluating LV contractility, offering an alternative to EF measurements. In particular, myocardial strain measurement utilizing cardiac MRI has been clinically adopted as a supplementary approach to EF for a more precise assessment of cardiac function.

Clinically, patients with IMR exhibit a wide range of coronary artery disease, mitral regurgitation, and cardiac function. Hence, investigating the true impact of volume overload on the ischemic heart is challenging. In our laboratory, we have established a swine model with inferolateral wall infarction by occluding the branches of the left circumflex artery just distal to the obtuse marginal branch through percutaneous ethanol injection (23). This animal model consistently produces infarctions of nearly identical size in the inferolateral wall, accompanied by a varying degree of mitral regurgitation, thereby enabling us to compare the burden of MR on the ischemic heart using cardiac MRI. However, the sample size of this study was limited, and moderate/severe MR group has a slightly larger extent of infarction, larger EDV and ESV, which could potentially contribute to a higher propensity for developing MR.

In this study, we assessed the temporal changes in cardiac strains in each region of infarcted hearts with either no/mild MR or moderate/severe MR using preclinical swine model. Our data demonstrated that LS and CS in the infarct zone and the border zone decreased in both groups at 2.5 months after MI compared to baseline. However, msMR group exhibited significantly elevated LS and CS in the remote zone at 2.5 months compared to baseline. We also assessed the correlation between EDV and cardiac strains in the remote zone as mentioned in the Result section. CS exhibited a weak correlation with EDV, while LS showed no correlation. This can be explained by the fact that LS is generally considered less volume-dependent (7). Although it is generally considered to be less susceptible to volume changes compared to EF, cardiac strain has been reported to vary with alterations in preload and afterload (24). Thus, meticulous attention is required for the accurate interpretation of these data. Ruppert et al. investigated the effects of preload and afterload on longitudinal strain and LV contractility using rodent models of pressure overload and volume overload. Their findings showed that in the pressure overload model, cardiac strain was predominantly influenced by afterload, whereas in the volume overload model, it was primarily determined by contractility. However, a strong correlation with ventriculo-arterial coupling was observed in both models, highlighting the significance of the relationship between contractility and afterload. Importantly, a reduction in contractility was already evident in the volume overload model, indicating that cardiac function was already within the non-compensatory phase (25). In our swine model, considering the compensatory phase, it is conceivable that loading conditions characterized by elevated preload and reduced afterload, with maintained LV contractility, may lead to augmented strains in the remote zone, presumed to be normal myocardium.

The presence of dyssynchrony can exacerbate IMR by affecting the timing and coordination of the ventricular contractions, leading to inefficient pumping and mitral valve function (26). Soyama et al. reported that dyssynchrony in myocardial segments adjacent to papillary muscles significantly contributed to MR, independent of traditional factors such as left ventricular enlargement and annular dilatation. This suggests that dyssynchrony might disturb the synchronized closure of mitral leaflets, thereby leading to the progression of MR (27). In our study, cardiac dyssynchrony was also observed in the msMR group, and was more pronounced in CS. Furthermore, some early elevated strains were noted in temporal strain changes of LS and CS during one cardiac cycle in the msMR group at 2.5 months compared to the nmMR group. These changes could serve as effective indicators of ventricular remodeling, facilitating the early detection of heart failure progression.

## Limitations

This study, like any preclinical animal study, has its limitations in some respects. The first limitation is the animal model used, which was developed by inducing lateral wall infarction through ethanol injection into the branches of the left circumflex artery. This methodology facilitated the creation of an adequately sized lateral wall infarction, encompassing a wide range of MR severity while maintaining a high survival rate. However, patients with IMR often exhibit a broad spectrum of coronary artery diseases, with the ischemic lesions on their myocardium being heterogeneous. The advantage of this animal model lies in its capability to enable a precise comparison of the myocardial response in each region to volume overload by generating myocardial infarctions of consistent size. Secondly, in this experiment, we followed the animals for 3 months after inducing MI. No significant difference in cardiac function was observed between the two groups during this period, indicating the animals remained within the compensatory phase of heart failure. With extended observation, additional strain alterations in both groups will be expected. Especially in the moderate/severe MR group, a decrease in strains in the remote zone will occur, signifying the onset of decompensation due to volume overload and adverse ventricular remodeling. Further investigation is warranted to comprehensively elucidate the temporal changes in regional strain that lead to the decompensated phase. Thirdly, heart rate control during cardiac MRI, commonly achieved through administering beta-blockers in clinical settings, was not applied. Furthermore, the imaging procedures required adjustments on a case-by-case basis due to anatomical and skeletal differences from humans, leading to variability in the visualizations of each chamber view and short-axis views. Moreover, limitations in image resolution significantly impacted strain analysis during the diastolic phase, necessitating the exclusion of diastolic phase strain changes from this study. Finally, it should be noted that the sample size in this study is relatively small, potentially limiting the generalizability of the findings. Therefore, further research with a larger sample size is necessary to enhance the robustness and applicability of the results.

## Conclusion

In conclusion, elevated longitudinal strain and circumferential strain in the remote zone were observed in moderate/severe MR and ventricular dyssynchrony. Further study is essential to investigate the long-term impact of these elevated strains and dyssynchrony on ventricular function and their role in the progression of MR and heart failure.

## Data Availability

The original contributions presented in the study are included in the article/**Supplementary Material**, further inquiries can be directed to the corresponding author.

## Supplementary material

Supplementary Figure S1 Comparison of LS at BL between nmMR and msMR. There are no differences. A, Time course of LS during end-diastole to end-systole in each chamber view. B, Peak strain in each chamber and mean of them. C, Peak strain in each zone. BL, baseline; LS, longitudinal strain; CH, chamber; IZ, infarction zone; BZ, border zone; RZ, remote zone.

Supplementary Figure S2 Comparison of CS at BL between nmMR and msMR. There are no differences. A, Time course of CS during one heart cycle in each chamber view. B, Peak strain in each chamber and mean of them. C, Peak strain in each zone. BL, baseline; CS, circumferential strain; Base; IZ, infarction zone; BZ, border zone; RZ, remote zone.
