# Supplementary figures and images for: Mitral regurgitation increases systolic strains in remote zone and worsens left ventricular dyssynchrony in a swine model of ischemic cardiomyopathy
Source: Front Cardiovasc Med. 2024 May 28;11:1397079. doi: 10.3389/fcvm.2024.1397079 (PMC11165204; doi:10.3389/fcvm.2024.1397079)

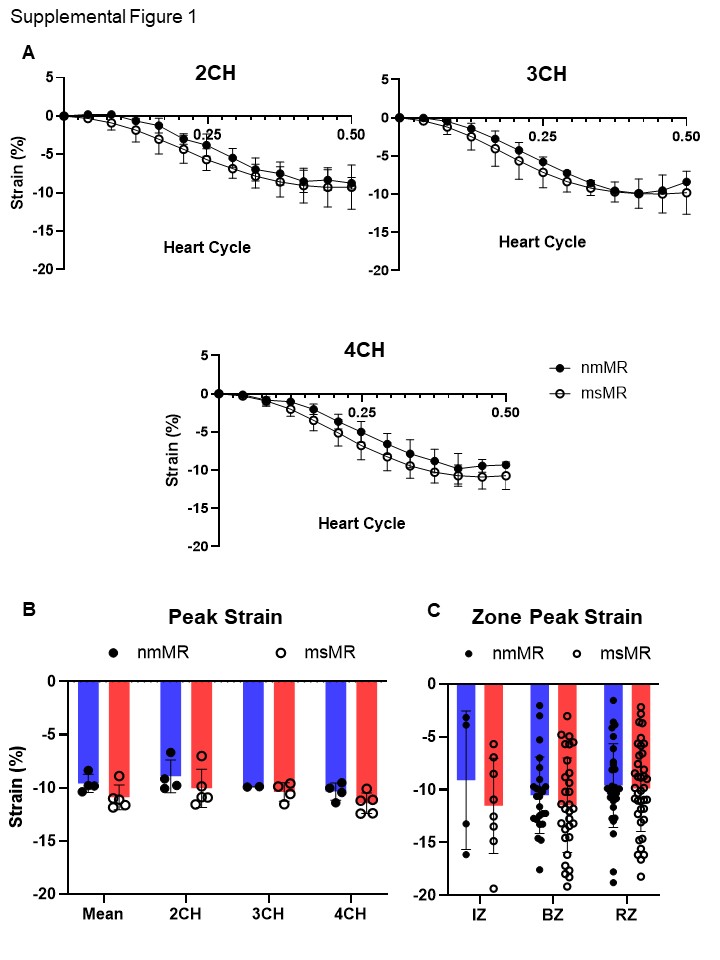

Supplement: Supplementary Figure S1 — Comparison of LS at BL between nmMR and msMR. There are no differences. A, Time course of LS during end-diastole to end-systole in each chamber view. B, Peak strain in each chamber and mean of them. C, Peak strain in each zone. BL, baseline; LS, longitudinal strain; CH, chamber; IZ, infarction zone; BZ, border zone; RZ, remote zone. [file Image1.jpeg]

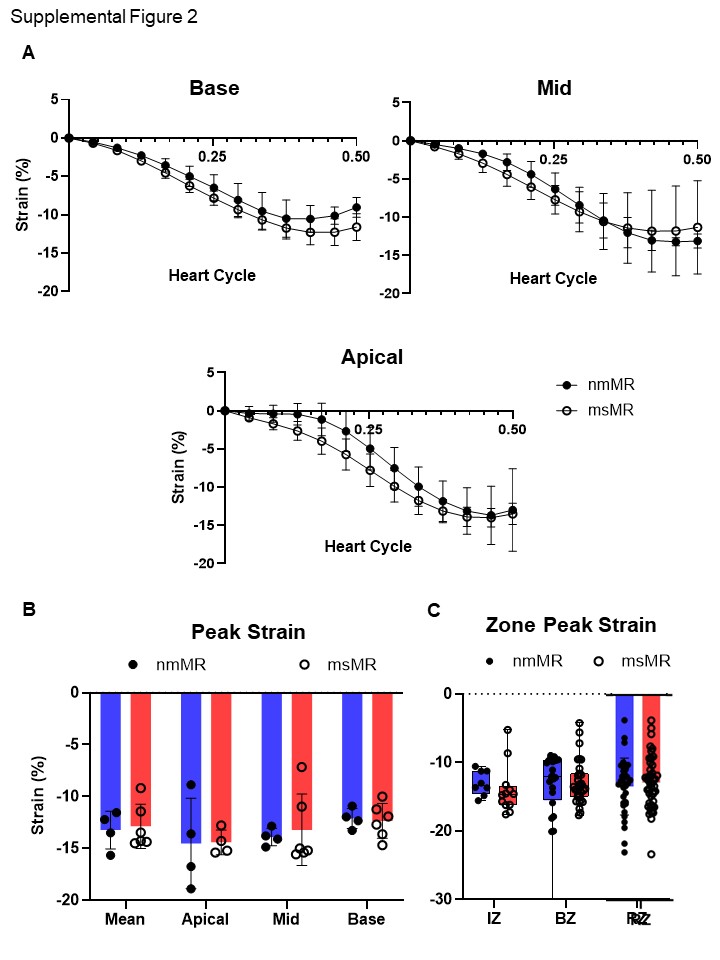

Supplement: Supplementary Figure S2 — Comparison of CS at BL between nmMR and msMR. There are no differences. A, Time course of CS during one heart cycle in each chamber view. B, Peak strain in each chamber and mean of them. C, Peak strain in each zone. BL, baseline; CS, circumferential strain; Base; IZ, infarction zone; BZ, border zone; RZ, remote zone. [file Image2.jpeg]
